# Catarrh of the Bladder in Old People

**Published:** 1880-07

**Authors:** 


Catarrh of the Bladder in Old People.—The important practical point in relation to treatment is first to ascertain the occasion of the local catarrh. In nine out of ten of these cases it consists in inability, often only to a slight extent, on the part of the patient to empty the bladder completely. The universally acknowledged cause, hypertrophy of the prostate, is, of course, the first in order of frequency. But after this are others not infrequent. Defective action may be due, first, to simple atony, the result of past habitual or occasional over-distension of the bladder with urine; secondly, to thickened and incompetent muscular paralysis of the bladder after chronic inflamation, sometimes associated with old stricture; thirdly, to defective innervation seen in connection with other slight signs of impaired function in a nervous centre, the last being, of course, the most serious of all, in its nature and probable results.

In all of these, local treatment, by carefully removing all the secretion by means of a soft catheter two or three times a day, perhaps aided by gently washing out the remainder, is the chief efficient remedy. Remember that this incompetence of the bladder is always to be sought for by physical examination; no other form of evidence in relation to it, as the patient's sensation, etc., is to be accepted as trustworthy. The introduction of a soft catheter immediately after the patient has passed water by his natural efforts, is the only test, and it should be applied on two or three occasions before arriving at a definite conclusion. The casual relation between the group of symptoms enumerated at the outset, and the defective function described, is far more common than it is generally supposed to be. It is on this account, therefore, that I have asked your attention specially to the subject.—*Sir Henry Thompson in London Lancet.*

